# Cervical Spondylotic Myelopathy: Factors in Choosing the Surgical Approach

**DOI:** 10.1155/2012/783762

**Published:** 2012-01-24

**Authors:** Praveen K. Yalamanchili, Michael J. Vives, Saad B. Chaudhary

**Affiliations:** Department of Orthopaedics, University of Medicine and Dentistry-New Jersey Medical School, 140 Bergen Street, Suite D1619, Newark, NJ 07103, USA

## Abstract

Cervical spondylotic myelopathy is a progressive disease and a common cause of acquired disability in the elderly. A variety of surgical interventions are available to halt or improve progression of the disease. Surgical options include anterior or posterior approaches with and without fusion. These include anterior cervical discectomy and fusion, anterior cervical corpectomy and fusion, cervical disc replacement, laminoplasty, laminectomy with and without fusion, and combined approaches. Recent investigation into the ideal approach has not found a clearly superior choice, but individual patient characteristics can guide treatment.

## 1. Introduction

Cervical degenerative disease, or cervical spondylosis, is an age-related change affecting the cervical spinal column. Radiographic evidence of cervical spondylosis can be found in 85% of individuals over sixty years of age [1]. Certain occupations and activities that place increased loads on the head may have a predisposition for cervical degenerative disease. Cervical myelopathy is a clinical syndrome that may result from cervical spondylosis. When cervical myelopathy is a result of spondylosis, it is referred to as cervical spondylotic myelopathy (CSM).

Cervical spondylotic myelopathy manifests as long-tract clinical findings in the upper and lower extremities caused by spinal cord compression [2]. Patients present with a variety of findings, including clumsiness, loss of manual dexterity, difficulty with gait or balance, urinary complaints, motor weakness, sensory changes, and abnormal or pathologic reflexes. Appropriate initial imaging of CSM consists of plain static radiographs and flexion extension views to evaluate for instability. The advancing imaging of choice is magnetic resonance imaging (MRI) of the cervical spine to evaluate the soft tissues about the spine and the spinal cord. Clinical correlation is important when evaluating MRI changes as MRI can be overly sensitive and reveal abnormalities in asymptomatic adults [3]. Electrodiagnostic studies may be helpful to exclude other causes of upper extremity symptoms, such as suspected peripheral nerve entrapment syndromes.

The natural history of CSM is a progression of symptoms in a stepwise fashion over time [4]. Patients with mild myelopathy (that does not interfere with function) may be offered a trial of nonoperative management, whereas progressive, long-standing, or severe myelopathy is candidates for surgical decompression of the spinal cord in the affected areas [5, 6]. Operative intervention may be via anterior, posterior, or combined approaches and with or without fusion. Anterior options include single or multilevel anterior cervical discectomy and fusion (ACDF), anterior cervical corpectomy and fusion (ACCF), and cervical disc replacement (CDR). Posterior options include laminectomy without fusion, laminectomy and instrumented fusion, and laminoplasty. Factors to consider when selecting the operative approach include location of cord compression, number of levels involved, sagittal alignment, instability, associated axial neck pain, and risk factors for pseudoarthrosis.

## 2. Anterior Surgical Options

**Figure 1:**
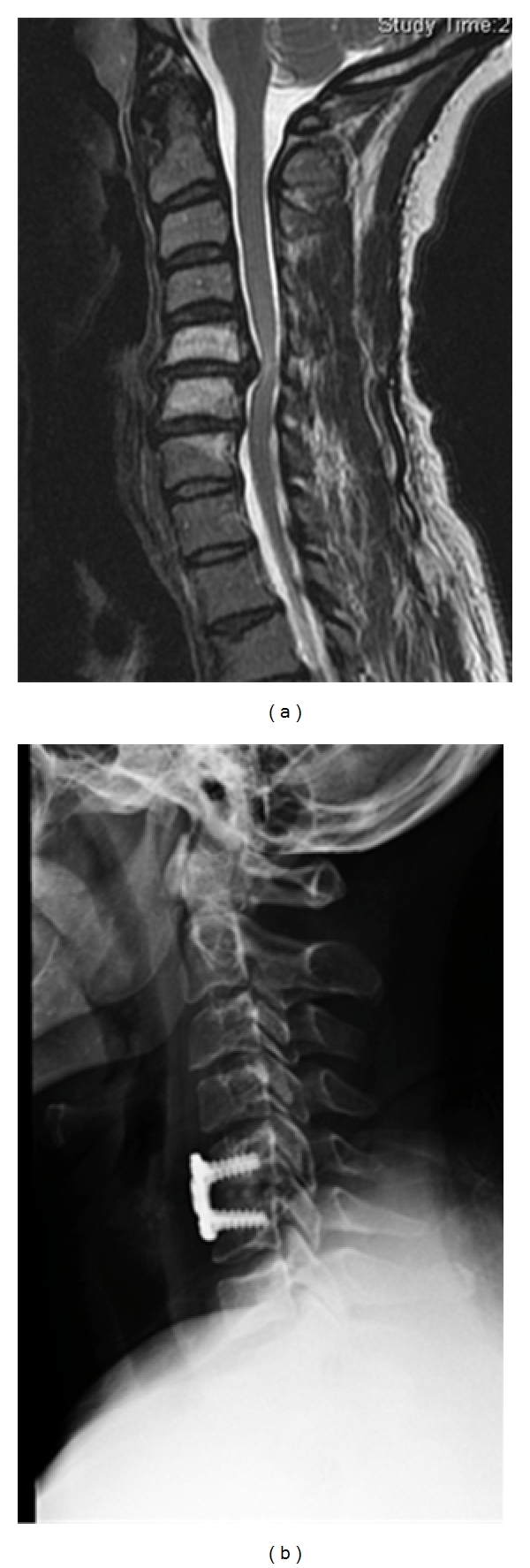
(a) Sagittal MRI demonstrating C5-6 extruded disc herniation. (b) Lateral radiograph of same patient after undergoing anterior cervical discectomy and fusion.

The anterior surgical options can be used for both single level and multilevel disease. The anterior approach is generally favored with soft disc herniations, concomitant severe axial neck pain, kyphosis, and with 1-2 levels of involvement (Figure 1).

**Figure 2:**
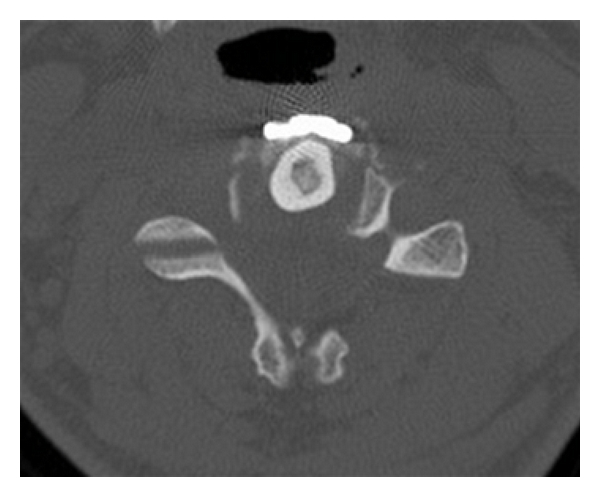
Axial CT scan demonstrating fibula strut graft placed in central corpectomy trough.

ACDF utilizes a Smith-Robinson approach to access the anterior surface of the cervical spine. After incision of the platysma, this approach involves little muscle disruption but opening of the pretracheal and prevertebral fascial planes to mobilize the midline structures of the neck. The decompression involves a thorough discectomy with removal of cartilaginous end plates and posterior osteophytes. A left-sided approach is preferred by some due to a more favorable course of the recurrent laryngeal nerve. Adequate decompression of the spinal cord may require removal of posterior osteophytes, partial corpectomy, or removal of the posterior longitudinal ligament (PLL); however all of these procedures increase the risk of injury to the spinal cord. ACCF is an alternative to multilevel ACDF and utilizes a similar approach, with either a transverse or longitudinal incision depending on number of levels. In this technique a central trough of vertebral body is progressively removed with a combination of a high-speed burr and rongeurs (Figure 2).

The trough is centered between the uncovertebral joints, which helps orient the trough over the spinal cord and ensure complete decompression. Care must be taken to avoid eccentric bone removal laterally, endangering the vertebral arteries. A thin shell of the remaining posterior wall and posterior longitudinal ligament can then be removed with microcurrettes and Kerrisons. Fusion with ACDF and ACCF may be achieved with various graft options, including autologous tricortical iliac crest graft, allograft, polyetheretheketone (PEEK), or metal cages or a combination of morsellized bone from the corpectomy plus a structural allograft or cage. Plating is now common, especially with multilevel ACDF and ACCF [7, 8]. Complications with the anterior approach include vertebral artery injury (0.3%), esophageal injury (0.2–0.4%), wound infection (0.2–1.4%), and dysphagia (28–57%) [9]. The cause of dysphagia appears to be multifactorial, including traction on the superior laryngeal nerve, pharyngeal plexus, recurrent laryngeal nerve, and esophageal retraction. Risk factors for dysphagia include age >60, multiple levels, revisions, females, thick plates, and longer preop pain [10].

**Figure 3:**
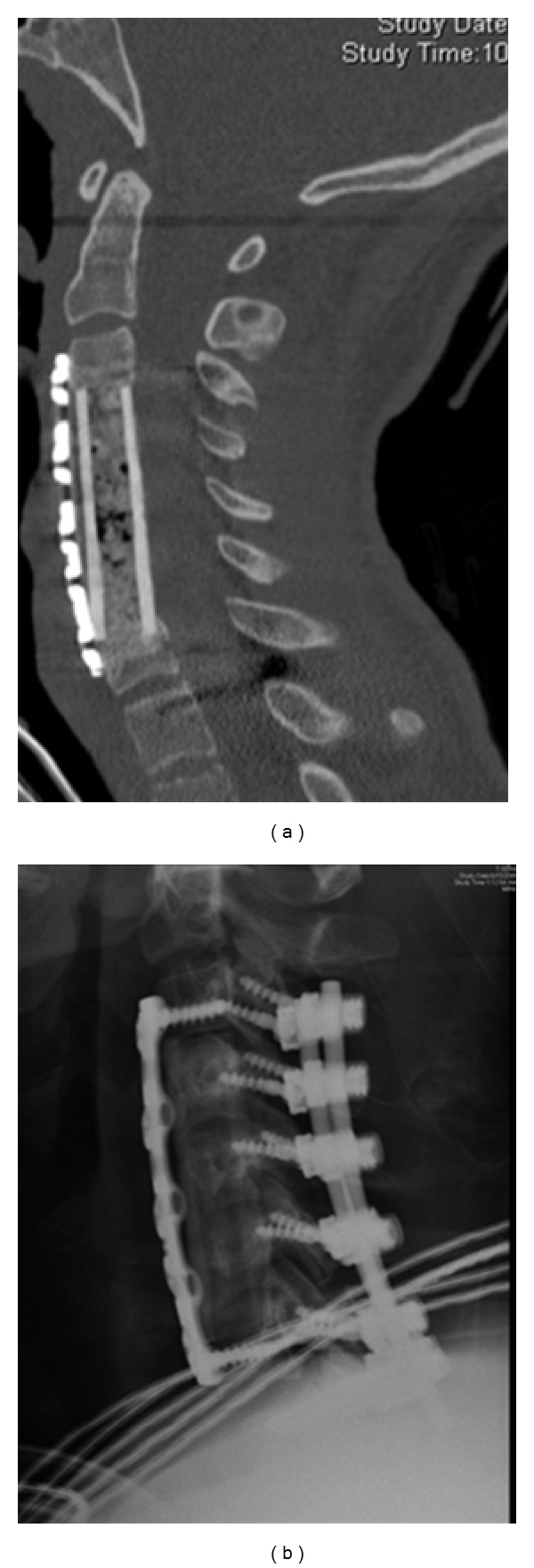
(a) Sagittal reconstructed CT scan of patient who underwent 3-level corpectomy. (b) Lateral radiograph of same patient. Posterior fusion was performed to increase stability of the construct.

Advantages of ACDF or ACCF include ability to directly decompress offending structures, decompress the anterior spinal artery, restore cervical lordosis, and address axial neck pain. Multilevel ACDF is preferred in certain situations over ACCF where the compression is confined to the level of the disc spaces. Also, it is associated with a less blood loss and has a lower risk of graft kick out and catastrophic failure [11]. However multilevel ACDF is associated with an increased risk of pseudarthrosis, as high as 54% in three-level fusions [12]. Some surgeons use off-label recombinant human bone morphogenetic protein-2 (rhBMP-2) in these situations, but this should be undertaken with caution as there have been reports of airway compromise due to swelling [13]. ACCF is preferred when compression extends behind the vertebral bodies to ensure that all areas of compression are addressed. When multilevel corpectomies are performed, there is potential for significant plate failure and graft extrusion, so supplemental posterior instrumentation should be considered [14] (Figure 3).

Some have suggested that a potential benefit of ACCF is that fewer graft surfaces are required to fuse than multilevel ACDF (i.e., for a decompression at C4-5/C5-6, ACDF would require 4 surfaces to fuse versus 2 surfaces if treated with ACCF). Multiple studies have compared the fusion rates of ACCF and ACDF in an attempt to verify this benefit. Nirala et al. investigated 201 patients with multilevel noninstrumented anterior fusion and found that with more levels ACCF had a higher fusion rate than ACDF [15]. Another study investigated 52 patients with multilevel anterior fusion with autograft and plate fixation and found similar clinical and fusion rates between ACCF and ACDF [16]. With modern plating techniques, it appears that fusion rates are similar between the two techniques [17]. A hybrid technique, combining selected corpectomies and discectomies, can be utilized where there is both retrodiscal and retrovertebral compression. Such a construct can increase stability and obviate the need for posterior supplementation. Shen et al. investigated the pseudarthrosis rate of multilevel anterior cervical fusion with rhBMP-2 and allograft using a hybrid technique in 127 patients [18]. Overall pseudarthrosis rate was 10%, with 4% for three levels, 17% for four levels, and 22% for five levels. Nonunions typically occurred at the lowest level.

CDR is another anterior option in cases where cord compression is confined to the retrodiscal region. As a nonfusion option, this may provide the theoretical benefit of decreasing adjacent segment degeneration. Buchowski et al. compared ACDF with CDR for myelopathy at a single level disc space [19]. These authors found similar improvement in neurologic status between the two groups at two years. Recently two-level CDR has come under investigation [20].

## 3. Posterior Surgical Options

The posterior surgical options are generally utilized for multilevel compression, such as in cases of congenital stenosis, older patients with advanced multilevel spondylosis, and certain cases of ossification of the posterior longitudinal ligament (OPLL) [21, 22]. The posterior approach relies on decompression through both direct removal of offending posterior structures and indirectly, through spinal cord translation posteriorly [23]. Therefore when spinal cord compression is from anterior structures, patients should have maintenance of lordosis or correctable kyphosis to permit adequate indirect decompression [24]. Posterior approaches utilize a midline approach through the posterior cervical skin and musculature followed by subperiosteal dissection of the selected levels. Extent of dissection laterally over the facets is dependent on whether a concomitant fusion is to be performed.

**Figure 4:**
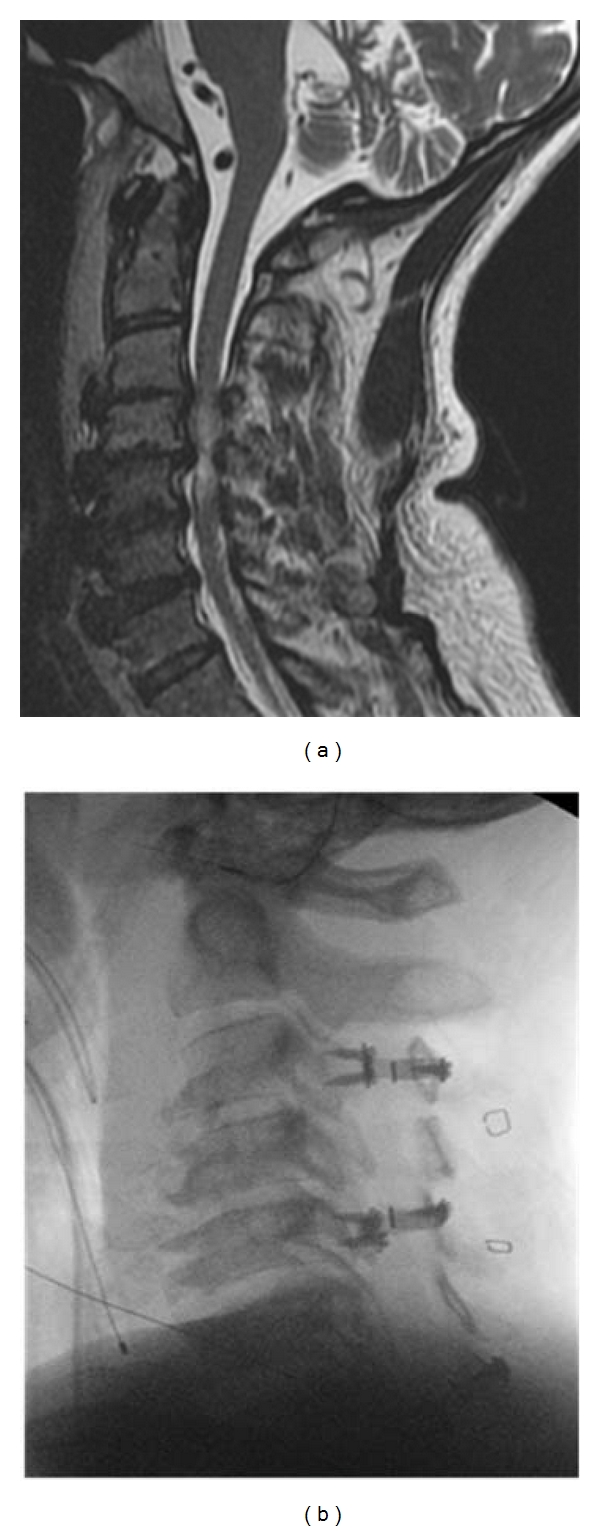
(a) Sagittal MRI of a patient with multilevel stenosis and preserved lordosis. (b) Lateral radiograph of same patient after canal-expanding laminoplasty.

Laminoplasty increases the effective diameter of the spinal canal while preserving the posterior elements of the cervical spine as a biologic covering over the spinal canal. Laminoplasty requires at least 10 degrees of lordosis to allow posterior shift of the spinal cord for indirect decompression [25]. In the open-door technique, two troughs are created at the junction of the lateral masses and lamina with the use of a high-speed burr. One side is completed with microcurrettes or Kerrison rongeurs, the other side left with a thin shell of bone that is then “greensticked” creating a hinge. Once opened, the door can be kept patent with a variety of techniques including suture or wiring of the spinous process to the facet joint, by insertion of a spacer within the opening, or with miniplate and screw fixation (Figure 4).

The main advantage of laminoplasty is the avoidance of fusion. Despite this, patients do experience decreased range of motion postoperatively of up to 50% [26]. Since fusion is not performed, the patient requires preexisting cervical stability, and upright and/or flexion-extension radiographs should be considered to confirm this preoperatively. Laminoplasty has been compared to corpectomy and laminectomy with fusion and has been shown to have similar clinical outcomes to both [27, 28]. Complications include C5 nerve root palsy, kyphosis, wound complications, and persistent or new axial neck pain [26, 29].

**Figure 5:**
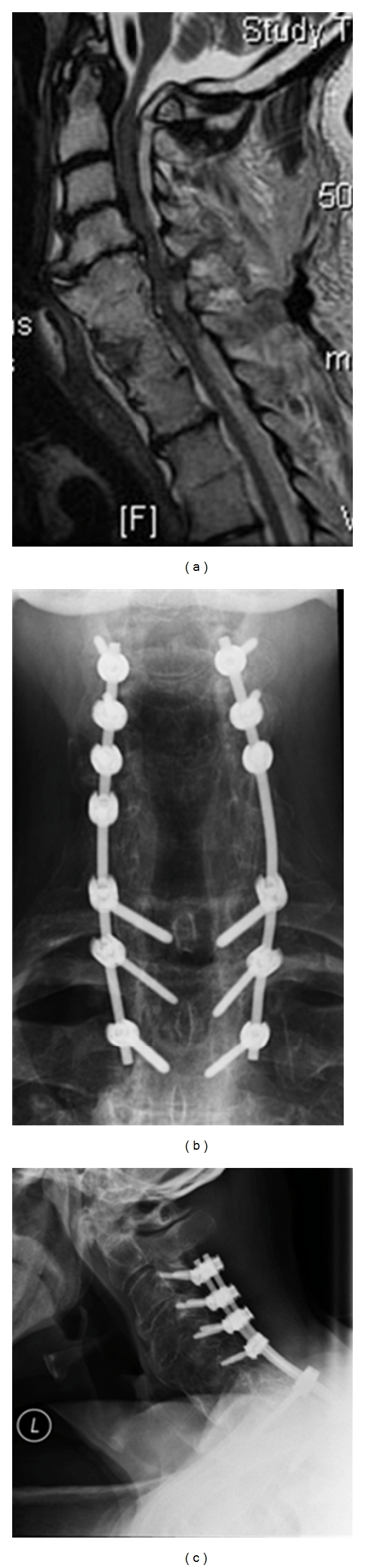
(a) Sagittal MRI of a patient with severe multilevel spondylosis and stenosis. (b-c) Anteroposterior (b) and lateral (c) radiographs of same patient after multilevel laminectomy and fusion. Note that distal fixation was achieved with pedicle screws which have increased pullout resistance compared with lateral mass fixation.

Laminectomy involves removal of the lamina and ligamentum flavum over the desired levels and can be performed with or without fusion and instrumentation. Laminectomy without fusion is generally restricted to patients with preserved lordosis who are poor candidates for fusion, since significant rates of progressive postoperative kyphosis have been reported [30, 31]. Instrumented fusion should be utilized for most cases, especially in circumstances of correctable kyphosis and instability. A multitude of instrumentation and screw techniques as well as graft choices exist and can be utilized at the discretion of the individual surgeon. Complications of multilevel laminectomy and fusion include C5 nerve root palsy, wound complications, and hardware failure [2]. In cases of long multilevel laminectomy and fusion, caudal fixation in the C7 lateral masses is suboptimal due to their small size. Pedicle screws at either C7 or the top 2 thoracic vertebrae decrease the chance of distal fixation failure in these long constructs (Figure 5).

With the aforementioned considerations in mind, the primary indication for a combined anterior and posterior approach is multilevel compression in the setting of fixed kyphosis, especially if 2 or more corpectomies must be performed. It can also be considered in patients with localized disease and poor bone quality or high risk for pseudarthrosis. Konya et al. reported on 40 patients treated with combined anterior and posterior approaches for CSM [32]. All patients had three- to four-level disease. At one-year follow-up neurologic function was improved in all patients with a 97.5% fusion rate with no reported instrumentation complications. The exact number of levels to consider combined approach is still debated.

## 4. Comparative Efficacy

Recently a systematic review sponsored by the American Association of Neurological Surgeons (AANS)/the Congress of Neurological Surgeons (CNS) was performed to develop evidence-based guidelines for choosing among the available surgical options for treatment of CSM [17]. The National Library of Medicine and Cochrane Databases were queried using MeSH headings and keyword regarding anterior and posterior surgery and CSM. An evidentiary table was assembled to summarize the quality of evidence from I to III (lowest). Recommendations were formulated containing degree of strength based on Scottish Intercollegiate Guidelines. Most of the manuscripts were found to be Class III. The results of the paper were that ACDF, ACCF, laminoplasty, laminectomy, and laminectomy with fusion all yielded similar near term functional improvements for CSM. Laminectomy without fusion, however, is associated with late deterioration. Another recent systematic review of retrospective cohort studies showed that ACCF, ACDF, laminoplasty, and laminectomy and fusion yielded similar neurologic recovery [33]. The major differences between the groups were the associated complications. Therefore it appears that, given the available literature, the choice of surgical approach will be more dependent on the individual patient factors described previously than the superiority of any one surgical option. This clinical equipoise has been the motivating factor for interest in pursuing a prospective randomized clinical trial and for the distinction of CSM as one of the national health research priorities for comparative effectiveness research by the Institute of Medicine (Medicine Io; Initial National Priorities for Comparative Effectiveness Research; http://www.iom.edu/. Accessed May 31, 2011).

## 5. Conclusions

Cervical spondylotic myelopathy is a progressive disease that often requires surgical intervention. A variety of surgical options exist, including anterior and posterior approaches with and without fusion. Evidence-based review has not clearly shown one technique to be clinically superior to another. Therefore decision-making will depend on individual patient factors and associated approach-related complications. Factors to consider include location of cord compression, number of levels involved, sagittal alignment, instability, associated axial neck pain, and risk factors for pseudoarthrosis.
